# Bite Reconstruction in the Aesthetic Zone Using One-Piece Bicortical Screw Implants

**DOI:** 10.1155/2018/4671482

**Published:** 2018-04-29

**Authors:** Stefan Ihde, Łukasz Pałka, Maciej Janeczek, Piotr Kosior, Jan Kiryk, Maciej Dobrzyński

**Affiliations:** ^1^Dental Implants Faculty, International Implant Foundation, 116 Leopold Street, 80802 Munich, Germany; ^2^Reg-Med Dental Clinic, Rzeszowska 2, 68-200 Żary, Poland; ^3^Department of Biostructure and Animal Physiology, Wroclaw University of Environmental and Life Sciences, Kożuchowska 1, 51-631 Wroclaw, Poland; ^4^Department of Conservative Dentistry and Pedodontics, Wroclaw Medical University, Krakowska 26, 50-425 Wroclaw, Poland; ^5^Private Dental Clinic Maciej Kozłowski, Spokojna 23, 56-400 Oleśnica, Poland

## Abstract

The aim of this article was to present the clinical application of a new, smooth surfaced one-piece bicortical screw implant with immediate loading protocol. An 18-year-old, healthy male patient with a history of total dislocation and replantation of teeth 11 and 21 in early childhood was admitted to the clinic. Teeth 11 and 21 were extracted, and two long one-piece implants were inserted at extraction sockets in one surgical session under local anesthesia. Temporary composite crowns were placed in the patient on the same day. After 3 months, the single-phase two-layer impression was made and the composite crowns were replaced with metal-ceramic crowns. After 12 months, satisfactory aesthetic and functional results were obtained.

## 1. Introduction

Recently, immediate implant placement after tooth extraction with early loading has become a more common procedure, especially when the anterior teeth are missing. The advantages of this procedure include fewer surgical interventions, reduction in overall treatment time, reduced soft and hard tissue loss, and psychological satisfaction to the patient.

The aim of this article was to present the clinical application of a new, smooth surface, one-piece bicortical screw implant with immediate loading protocol. With its new design, it is now very simple to achieve durable reconstruction of function and a very good aesthetics [1]. The implant neck is bendable and the head can be ground, so there are no complications regarding the parallelism of the abutments. The paper reports the successful clinical case of immediate replacement of two frontal incisors with active fistula and periodontitis. The complete treatment was conducted without bone or soft tissue augmentations and with minimal risk of peri-implantitis.

## 2. Case Report

**Figure 1:**
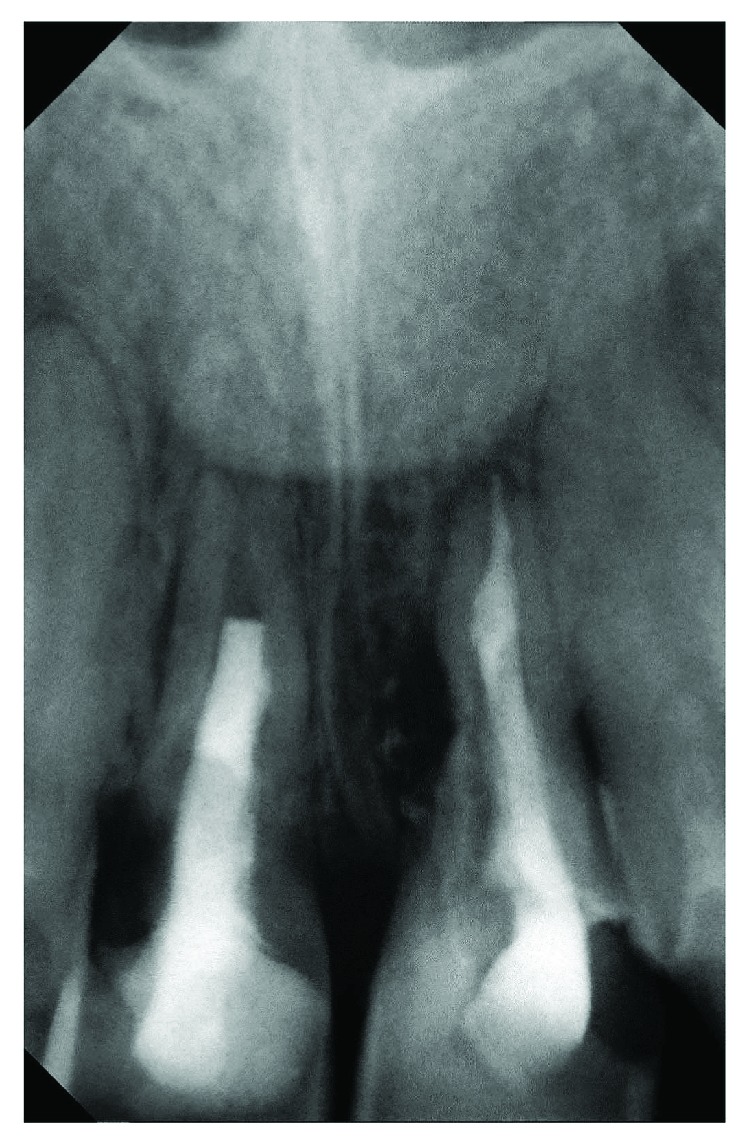
X-ray presenting severe root resorption of teeth 11 and 21.

**Figure 2:**
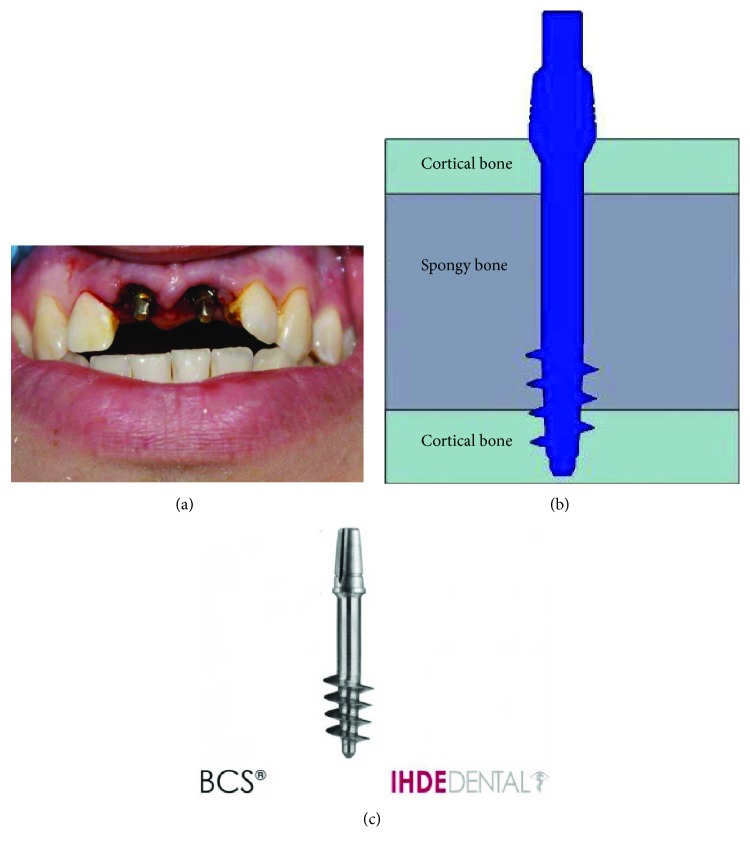
Immediate implant placement after tooth extractions: (a) the photo of immediate implants in the mouth right after placement, (b) the scheme of immediate implant placement, and (c) the photo of immediate implant.

**Figure 3:**
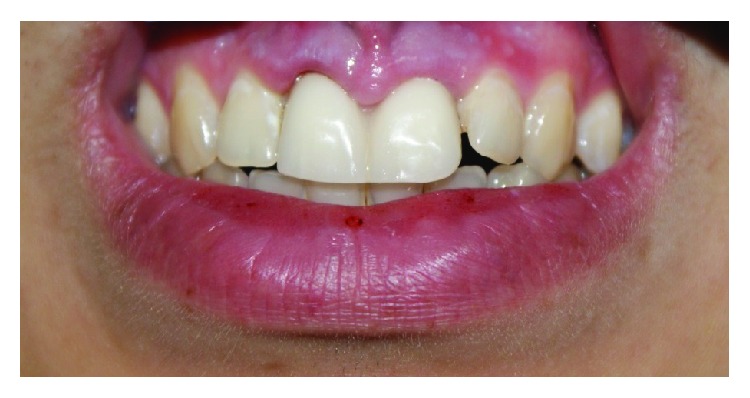
Temporary composite crown delivered on the same day. Still visible active fistula over tooth 11.

An 18-year-old, healthy male patient with a history of total dislocation and replantation of teeth number 11 and 21 in early childhood was reported to the clinic. Due to heavy root resorption, active fistula, and severe atrophy of the alveolar ridge, mostly related to the vestibular cortical plate, a decision was made to extract the teeth and to use one-piece immediate loading smooth surface bicortical screw implants (Figure 1) [2–4]. Following soft tissue cleaning with antiseptic 5% Betadine® solution, teeth 11 and 21 were extracted under local anesthesia (citocartin 100 solution and articaine 4% with Adrenaline 1 : 100000). The procedure was performed atraumatically with the careful use of luxators (SDI®) and periotomes (Medessa®) to avoid damage of the continuity of the alveolar ridge. Extraction sockets were thoroughly debrided and granulation tissue removed. The edges of the gingival garlands were aligned using a scalpel. The preparation of osteotomy sites was carried out using the sequential order of calibrated drills recommended by the manufacturer, cooled with saline solution in external mode at a speed of 800 rpm. The implant beds were prepared with the use of a 2.0 mm drill (30 mm long) on a straight handpiece. Two long one-piece implants with a diameter of 3.5 and a length of 22 mm were placed and anchored in the second cortical in the floor of the nose with a perfect primary stability (Figures 2(a)–2(c)) [5]. The implants were inserted into the bone (with insertion torque of 35–40 Ncm) using hand tools to achieve primary stabilization. Postoperative intraoral periapical radiograph was taken, confirming the accuracy of placement of implants. The extraction socket and space between the implant and the bone was filled with collagen sponge (Spongostan). Abutments were attached to the implant body and prepared for parallelism and adequate space. At the same day, provisional composite crowns were placed in the patient for immediate replacement of the missing front teeth due to functional and aesthetic requirements (Figure 3) [6].

**Figure 4:**
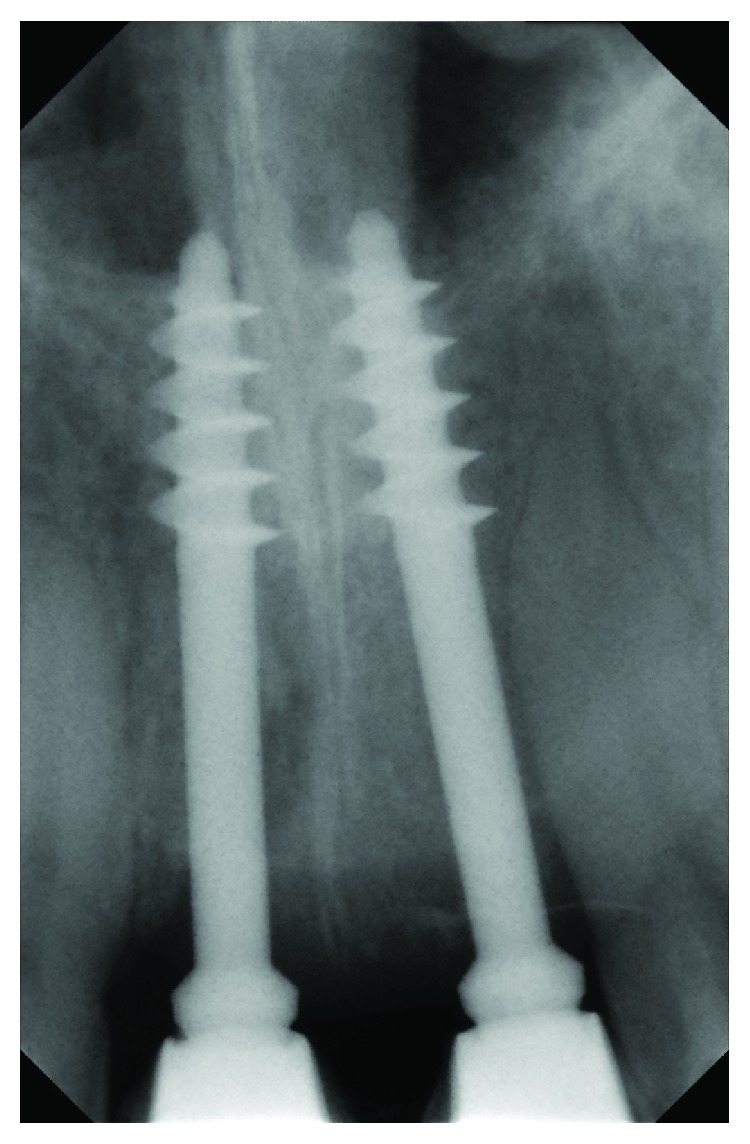
X-ray after 3 months with try-in metal-ceramic crowns.

**Figure 5:**
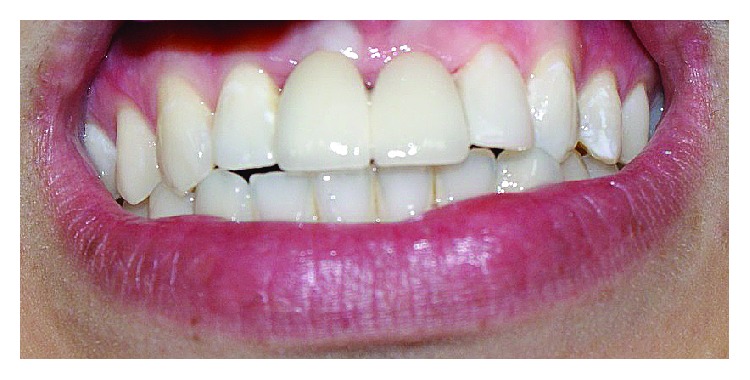
Metal ceramic crowns after cementation.

**Figure 6:**
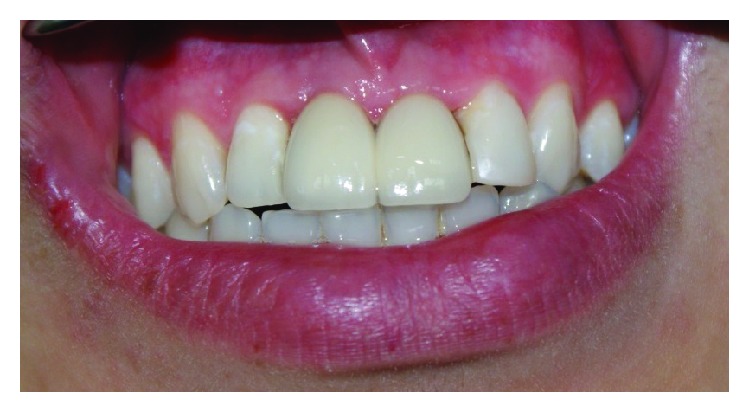
Good aesthetic after 12 months with papilla preservation.

After 3 months, when the peri-implant tissues have healed, the single-phase two-layered impression (Panasel transfer polyvinyl siloxane mass, Kettenbach®) of implant transfers was made with closed tray technique. The composite crowns were replaced with metal-ceramic crowns and cemented with Fuji IX cement (Figures 4 and 5) [7]. Follow-up was done after 3-, 6-, and 12-month intervals. Comparison of pre- and postprocedure radiographs clearly revealed elevated peri-implant marginal bone in response to the action of loading forces [8, 9]. Very good aesthetic result of this treatment was achieved by the preservation of gingival papillae (Figure 6).

## 3. Discussion

If chosen, in presented clinical case, conventional two-stage implantation (with or without immediate implant placement) would require bone augmentation following teeth extraction. The aforementioned implant can be loaded after a minimum of 6 to 9 months. The main drawback of this treatment option is lack of predicting the bone modeling process after extraction as well as implant placement. The use of biomaterials for bone regeneration is highly risky because of ongoing active inflammation; it is time-consuming and expensive. Thinking of the long-term outcome, we particularly need to consider the possible direction of physiological atrophy of the maxillary alveolar bone and complications in a case of implant surface exposure and its subsequent bacterial contamination [2].

The bicortical implant anchorage is an excellent treatment option, which allows us to predict the outcome of our treatment due to an anatomically stable position of at least one cortical bone (second or third). This type of bone does not undergo typical physiological changes and if damaged, its continuity will always be restored. The thin neck, penetrating the mucosa and polished surface of an implant, prevents bacterial contamination and peri-implantitis [2, 3, 10]. The transmission of load on the thread anchorage in the highly mineralized bone allows bone regeneration and (what is reserved only for bicortical, polished implants) regaining stabilization in case of sterile loosening of the implant. Even in case of bone resorption around the implant, it is possible to cutoff the head and replace it with a newly cemented abutment on the remaining implant neck.

According to the comparative studies of implantation using bicortical screws and two-phase implants (Integral Systems), patients experienced less postoperative discomfort in the case of the bicortical screws (less invasive treatment and no preparation of the periosteal flap) [11]. In clinical cases which did not require teeth extractions, patients were immediately provided (up to 3 days from the implantation procedure) with final prosthetic restorations of all types available on the market (metal-ceramic, metal-composite, and zirconium).

## 4. Conclusions

Clinical and radiographic evaluations after 12 months showed satisfactory preservation of marginal bone structure and peri-implant soft tissues condition as well as excellent aesthetic rehabilitation which is highly accepted by the patient. Our study revealed that final results and long-term success of immediately loaded one-piece implants were not different from conventional two-stage screw implants. The unquestionable benefits of bicortical implant use are the reduction of the number of visits, nonexistent need for regeneration procedures (and consequently costs reduction), and the possibility of immediate loading of the implanted screws due to optimal primary stabilization, which is obtained by placing the implants in the cortical bone. Regenerative procedures (including simultaneous implantation) are not required for this type of implants, but they can be implemented due to aesthetic or functional purposes. Despite the advantages of the use of bicortical implants allowing to achieve the immediate aesthetic rehabilitation, the risk of gingival recession and bone atrophy still exists. A sine qua non condition for a long-term prosthetic reconstruction based on bicortical implants, as well as for other types of restorations, is very good oral hygiene [3].
